# Evidence of Epstein-Barr Virus Association with Gastric Cancer and Non-Atrophic Gastritis

**DOI:** 10.3390/v6010301

**Published:** 2014-01-20

**Authors:** Juan L.E. Martínez-López, Javier Torres, Margarita Camorlinga-Ponce, Alejandra Mantilla, Yelda A. Leal, Ezequiel M. Fuentes-Pananá

**Affiliations:** 1 Virology and Cancer Research Unit, Federico Gomez Children’s Hospital of Mexico, Dr. Marquez No.162, Col. Doctores, Cuauhtemoc, Mexico City D.F. 06720, Mexico; E-Mail: jle_martinez@yahoo.com.mx (J.L.M.-L.); 2 Graduate Program in Biological Sciences, National Autonomous University of Mexico (UNAM), Av. Universidad Nº 3000, Mexico City D.F. 04510, Mexico; 3 Unit of Medical Research in Infectious and Parasitic Diseases (UIMEIP), Pediatric Hospital, 21st Century Medical Center, Social Security Mexican Institute (IMSS). Av. Cuauhtemoc 330, Col. Doctores, Cuauhtemoc, Mexico City D.F. 06720, Mexico; E-Mails: jtorresl57@yahoo.com.mx (J.T.); margaritacamorlinga@yahoo.com (M.C.-P.); 4 Oncology Hospital, 21st Century Medical Center, Social Security Mexican Institute (IMSS), Av. Cuauhtemoc 330, Col. Doctores, Cuauhtemoc, Mexico City D.F. 06720, Mexico; E-Mail: alemantimora@yahoo.com.mx; 5 Merida’s Medical Unit of High Specialty, Social Security Mexican Institute (IMSS), Merida, Yucatan, Mexico City D.F. 97150, Mexico; E-Mail: yeldaleal@gmail.com

**Keywords:** Epstein-Barr virus, EBV, gastric cancer, non-atrophic gastritis, cribriform pattern and lace pattern

## Abstract

Different lines of evidence support an association between Epstein-Barr virus (EBV) and gastric cancer (GC). The main understood risk factor to develop GC is infection by *Helicobacter pylori* (*H. pylori*), which triggers a local inflammatory response critical for progression from gastritis to GC. The role of EBV in early inflammatory gastric lesions has been poorly studied. A recent study proposed a cutoff value of 2000 EBV particles to identify patients with increased chances of infection of the gastric epithelium, which may favor the inflammatory process. To better understand the role of EBV in cancer progression, we analyzed 75 samples of GC, 147 control samples of non-tumor gastric tissue derived from GC patients and 75 biopsies from patients with non-atrophic gastritis (NAG). A first-round PCR was used for EBV detection in tumor and non-tumor controls and a more sensitive nested PCR for gastritis samples; both PCRs had lower detection limits above the proposed cutoff value. With this strategy 10.67% of GC, 1.3% of non-tumor controls and 8% of gastritis samples were found positive. An EBER1 *in situ* hybridization showed EBV infection of epithelial cells in GC and in a third of NAG samples, while in the other NAGs infection was restricted to the mononuclear cell infiltrate. EBV-positive GCs were enriched in lace and cribriform patterns, while these rare patterns were not observed in EBV negative samples. Our results support a role for EBV in GC and early precursor lesions, either as directly oncogenic infecting epithelial cells or indirectly as an inflammatory trigger.

## 1. Introduction

Gastric cancer (GC) is the second cause of cancer related mortality worldwide [1]. There are two main histological types of gastric adenocarcinoma based on Lauren’s classification: intestinal and diffuse [2]. Intestinal GC is a malignancy preceded by inflammatory lesions of increased severity: the earliest gastric lesion is a non-atrophic gastritis (NAG), which evolves to an atrophic gastritis (AG), intestinal metaplasia, dysplasia and GC; this sequential progression is recognized as the Correa sequence [3]. The diffuse type of GC does not follow the Correa sequence; although, it is accepted that a chronic NAG is also a major risk for this type of GC [4]. Infection of the gastric mucosa by *Helicobacter pylori* (*H. pylori*) is considered the main risk factor for intestinal and diffuse GC, and also for chronic inflammatory responses triggering tissue damage [5].

Epstein-Barr Virus (EBV) infection has been associated with several types of lymphoma and nasopharyngeal carcinoma (NPC) [6]. More than 80% of lymphoepithelioma-like (LEL) GCs, a rare GC histologically similar to NPC, and about 10% of other common types of GCs are also positive for EBV [7,8]. EBV chronically infects about 95% of the adult population, with virus usually present in B cells in a latent stage. Infected B cells circulating close to the upper gastro-intestinal (GI) tract sometimes switch to the lytic cycle producing viral particles that can be transmitted to other hosts. These intermittent episodes of viral reactivation favor infection of epithelial cells and are critical for triggering NPC [6]. Although, viral reactivation would also favor a local inflammatory response, the role of EBV in early inflammatory lesions remains poorly studied. We recently analyzed 333 children with recurrent abdominal pain, finding that the presence of EBV confers a four- to six-fold higher risk to present with severe inflammatory infiltrate compared to children infected only by *H. pylori* [9]. 

A recent study found that gastric tissues in which EBV infects epithelial cells present 3000-fold more viral particles than tissues with only infected B cells [10]. The authors proposed a cutoff value of 2,000 EBV particles/10^5^ cells to distinguish gastric epithelial cell infection, a requirement fulfilled for US and Honduras samples [10]. The aim of this study was to address EBV infection in clinical samples from patients with GC and NAG. Two PCR protocols were designed: a first round PCR was used to search for EBV in tissues from cancer patients, and a more sensitive nested PCR (15 additional amplification cycles) for NAG samples. Both PCRs had detection limits above the proposed cutoff value (>62-fold and >2-fold higher). Patients were recruited from two cities of high GC incidence in Mexico: Mexico City and Merida Yucatan, which are located in the center and South East of the country, respectively, and represent areas of mostly urban (Mexico City, Mexico) and rural (Merida, Mexico) population. Looking to better understand the role of EBV in the most common types of GC, GCs highly associated with EBV (LEL and stump or remnant GC) were not included in the study. EBV was detected in 10.67% of GC samples, 1.36% of samples from non-tumor control tissues and in 8% of samples with NAG. An EBER1 in situ hybridization confirmed epithelial cell infection in GC and in one third of NAG samples. EBV was restricted to lymphoid cells in the other NAG samples, in spite of evidence of high viral loads. These data provides further support for an etiologic role of EBV in GC, likely starting in early inflammatory lesions.

## 2. Results and Discussion

### 2.1. Sample Description

75 GC samples were analyzed, 44 were diffuse, 27 were intestinal and 4 were of a mixed type. Non-tumor controls were obtained from 147 GC patients; tissue was taken at least 2 cm apart from the tumor mass. In the gastritis samples the morphology of the epithelial glands was not atypical and superficial degenerative changes were observed in only a few samples; therefore, all gastritis were classified as non-atrophic (NAG). It has been documented that *H. pylori* tends to be absent in tumor microenvironments [11]; in agreement, less than 20% of the tumor samples showed bacterial colonization, whereas *H. pylori* was observed in 64% of the NAG biopsies. 19 biopsies from gastric bypasses were also included as a reference of patients with obesity-related inflammation, but without gastric symptoms. Four of the bypass biopsies were positive for *H. pylori* colonization, two were positive for follicular gastritis correlating with *H. pylori* presence, and one was positive for metaplastic changes (see Appendix Table A1).

### 2.2. PCR Detection Limits

**Figure 1:**
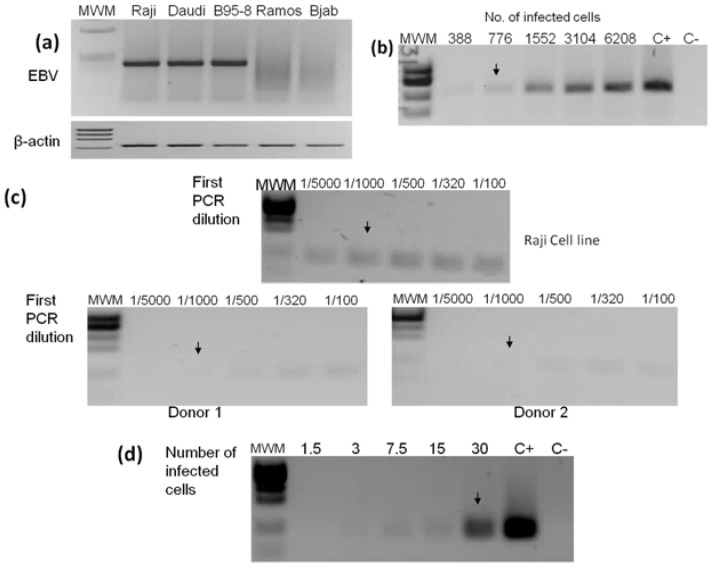
Standardization of EBV detection by first and nested PCRs. (**a**) Three positive and two EBV negative cell lines were used to standardize the PCRs. The viral 236 bp (first PCR) and 670 bp β-actin endogenous gene amplification products are shown; (**b**) DNA from Raji cells was serially diluted to find the first PCR lower limit of detection, which was established in 776 cellular genomes (black arrow); (**c**) Standardization of nested PCR. A 1/1,000 dilution of the first PCR product (upper panel) together with 15 additional amplification cycles were chosen to establish a condition in which EBV is not detectable by the nested PCR from DNA isolated from an equal number of peripheral blood leukocytes (an example of two EBV positive healthy donors is shown in lower panels); (**d**) The limit of detection for the nested PCR was established at 30 Raji genomes. The nested PCR amplicon is 104 bp. Raji and Ramos DNAs were used as positive (C+) and negative (C−) controls, respectively. MWM: molecular weight marker.

The first and nested PCRs were standardized using EBV positive and negative cell lines (Figure 1a). Raji cells were used to establish the limits of detection, a cell line carrying 50 EBV genomes per cell [12]. The lower limits of detection were 776 and 30 infected cells from an input DNA equivalent to 31,000 cellular genomes (Figure 1b). Thus, the number of EBV genomic copies detected by the first and nested PCRs is ≥38,800 and ≥1,500, respectively. Similar detection limits were obtained with B95-8 and Daudi cell lines (data not shown). About 50 EBV copies per cell have been reported in EBV positive lymphomas [13]. All tumor samples included in this study had between 5% to 80% tumor cells (mean = 43%). We reasoned that the first PCR should be sufficient to detect the virus if all tumor cells in GC samples were infected.

For NAG cases we cannot predict the frequency of EBV infected cells and a more sensitive nested PCR was implemented. Looking to increase the probability of detecting samples with epithelial cell infection, the limit of detection of the nested PCR was set up above the EBV load found in infected peripheral blood leukocytes (Figure 1c,d). Furthermore, the nested PCR detects viral loads of 0.048 particles/cell, twice above the viral load found in GC tissues in which EBV infection is restricted to B cells [10]. 

### 2.3. Detection of EBV in Gastric Cancer

**Figure 2:**
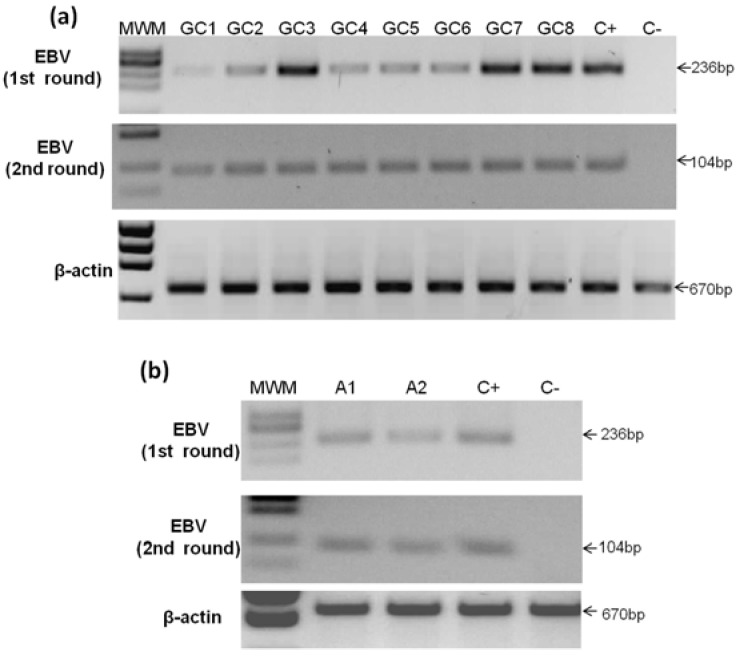
Analyses of gastric cancer and non-tumor control samples. (**a**) Eight samples were positive to EBV by first round PCR (upper panel) and confirmed by nested PCR (middle panel); (**b**) Two samples were positive to EBV in non-tumor control tissues by first round PCR (upper panel) and also confirmed by nested PCR (middle panel). DNA from Daudi cells was used as positive control (C+) and from Ramos cells as negative control (C−). MWM, molecular weight marker.

Seventy-five patients with GC were analyzed by the first PCR, and eight samples gave positive results, corresponding to 10.67% (Figure 2a and Table 1). In contrast, of the 147 cases of non-tumor controls, only two samples resulted EBV positive (1.36%) (Figure 2b). All positive GC and control samples were confirmed by nested PCR. We estimated the odds ratio (OR) of EBV in tumor *vs.* non-tumor control tissues, finding an OR of 8.66 (95% IC 1.8–41.9; p = 0.003), indicating a significantly increased chance to find EBV infection in tumors. 

All EBV negative GC and non-tumor control samples were subjected to nested PCR, and three additional GC and six control samples were found positive (data not shown). Since detection by nested PCR does not match the frequency of infected cells with the frequency of tumor cells, only the first PCR supports that infection preceded transformation. Both positive samples by the first PCR and three by the nested PCR of the non-tumor control samples corresponded to tissues in which the paired GC sample was positive to EBV by the first PCR.

**Table 1:** Characteristics of the EBV-positive GC samples.

No sample	Lauren	WHO	Other histological patterns	Lymphocyte infiltrate −/+++	Age	Gender	% of tumoral cells	Site
GC1	Mixed (Diffuse and intestinal)	Tubular Mucinous	Lace pattern with clear cells (LEL-like)	+++	87	Male	80	Body
GC2	Intestinal	Tubular	Cribriform	+	78	Female	10	Body and Antrum
GC3	Diffuse	Signet ring cells	None	++	38	Male	30	Body and Antrum
GC4	Intestinal	Tubular	Cribriform with clear cells	+	72	Female	60	Body
GC5	Diffuse	Signet ring cells	None	+	74	Female	70	Antrum
GC6	Intestinal	Tubular	None	+	72	Male	25	Body
GC7	Intestinal	Tubular	Cribriform	++	52	Female	60	Body and Antrum
GC8	Intestinal	Tubular	Lace pattern	+	57	Male	20	Antrum

GC samples were classified under the Lauren’s and WHO’s criteria; additional GC patterns are listed when present. Lymphocytic infiltration is indicated as + mild, ++ moderate and +++ severe.

### 2.4. Detection of EBV in Non-Atrophic Gastritis

**Figure 3:**
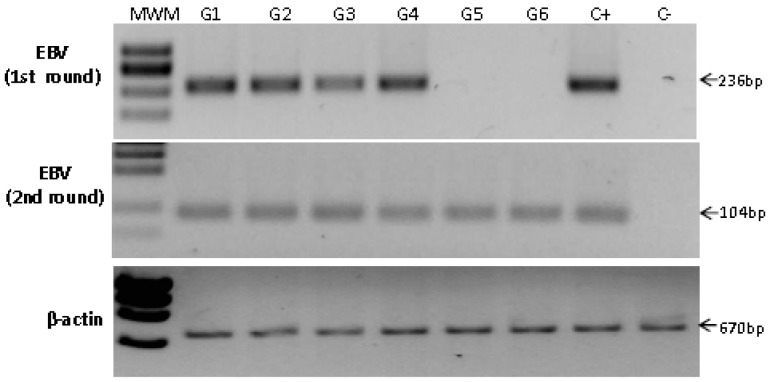
Analysis of non-atrophic gastritis samples. Four samples were positive for EBV by first round PCR (upper panel) and two additional samples gave positive results by nested PCR (samples G5 and G6, middle panel). DNA from Daudi and Ramos cells was used as positive (C+) and negative (C−) controls, respectively. MWM: molecular weight marker.

NAG is the earliest stage in the inflammatory pathway leading to both intestinal and diffuse GC. We hypothesized that if EBV were an important inflammatory trigger, similar to *H. pylori*, viral loads supporting EBV-infected epithelial cells would already be present in NAG samples. Seventy-five NAG samples were analyzed finding four positive samples by the first PCR and two by the nested PCR, jointly corresponding to 8% of the NAGs analyzed (Figure 3 and Table 2). 

**Table 2:** Characteristics of EBV-positive NAG samples.

Sample	Age	Gender	Inflammation −/+++	Activity −/+++	Site	Hp	PCR	ISH signal
G1	35	Male	++	++	Body	−	First	Lymphoid infiltrate
G2	25	Male	+++	++	Body	+	First	Lymphoid infiltrate
G3	64	Male	++	++	Body	−	First	Epithelial cell
G4	61	Female	+++	−	Antrum	+	First	Lymphoid infiltrate
G5	44	Female	+++	−	Antrum	NA	Nested	Epithelial cell & Lymphoid infiltrate
G6	23	Male	+++	−	Antrum	+	Nested	Lymphoid infiltrate

NAG samples were classified according to the Sydney system. Inflammation (mononuclear cell) and activity (polymorphonuclear cell) infiltration are indicated as − = none, + = mild, ++ = moderate and +++ = severe. NA = Not available.

### 2.5. Detection of EBV in Samples from Gastric Bypass Surgeries

**Figure 4:**
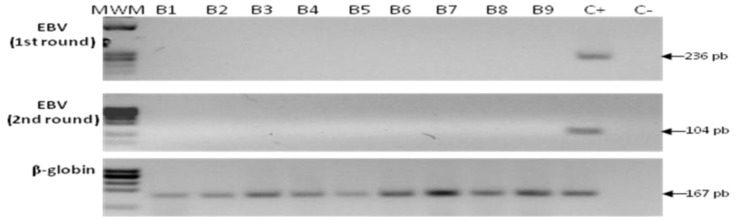
Analysis of samples from gastric bypass surgery. None of the bypass samples were positive for EBV; an example of nine of them is shown after the first round PCR (upper panel) and nested PCR (middle panel). DNA from Raji and Ramos cells was used as positive (C+) and negative (C−) controls, respectively. MWM: molecular weight marker.

A set of 19 samples from patients that were subjected to gastric bypass surgery was included in the study. Although, these individuals presented increased local inflammation (Appendix Table A1), this inflammation was related to obesity and none of the patients referred any symptom of gastric malaise. We reasoned that it was likely that these individuals had EBV infected B cells in the gastric inflammatory infiltrate; still the level of infection would be undetected by both PCRs. All 19 samples were negative for EBV by the first and nested PCRs (Figure 4), in spite of finding two patients with follicular gastritis and one patient with metaplastic changes. Of note, both gastric tissues showing lymphoid nodules formation were also positive for *H. pylori* colonization (Appendix Table A1). 

### 2.6. Sequencing

Both strands of the PCR products from all positive samples were sequenced and results were blasted at the NCBI web site. Sequences of the amplified PCR products presented at least 99% homology with EBV strains (AG876; accession number DQ279927.1 and B95-8; M15973.1). We observed four samples with single nucleotide changes arguing against a possible common source of viral contamination (data not shown).

### 2.7. *In situ* Hybridization (ISH) and Immunohistochemistry (IHC)

**Figure 5:**
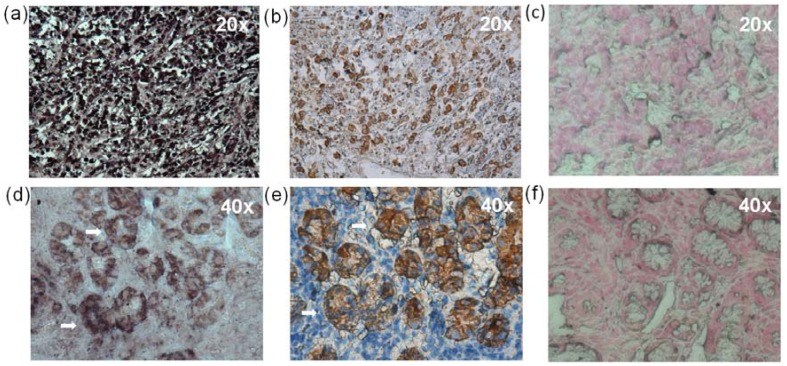
EBER1 and cytokeratin staining of EBV positive and negative GC and NAG samples. (**a**) EBER1 and (**b**) cytokeratin staining of an EBV positive diffuse GC sample (GC5 of Table 1), (**c**) EBER1 staining of an EBV negative diffuse GC sample. (**d**) EBER1 and (**e**) cytokeratin staining of an EBV positive NAG sample (G3 of Table 1), (**f**) EBER1 staining of an EBV negative NAG sample.

**Figure A1:**
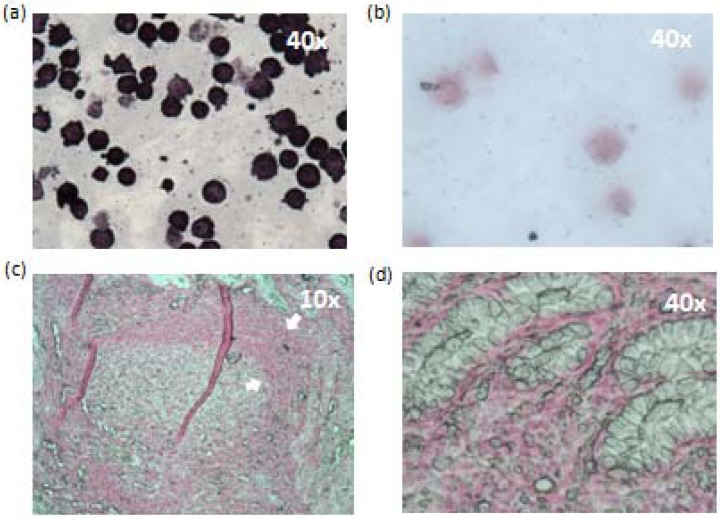
EBER1 ISH of controls and a bypass sample. (**a**) Raji cells were used as positive control and (**b**) MCF-10A cells were used as a negative control; (**c**) Gastric bypass sample with a follicular gastritis, arrows point out to a lymphoid nodule negative to EBER1; (**d**) Shows a larger magnification of the cells in the lymphoid nodule.

EBER1 *in situ* hybridization (ISH) was performed to test whether the EBV positive signal was originated from epithelial or B cells. The presence of EBV in GC tissues has been previously documented [7,8,14,15,16,17,18,19,20], and as such, of four EBV positive GC tissues tested, all four showed a positive signal in most or all of the gastric tumor cells (Figure 5a). EBV infection in NAG samples have been poorly explored, and since it is uncertain whether infected/damaged cells are already undergoing limited rounds of clonal expansion, and since a more sensitive PCR was used as criteria for positivity, it is unclear whether in GC precursor lesions there are already infected epithelial cells. On the other hand, it is possible that EBV infected B cells may be the target of the inflammatory response triggering NAG. When EBER1 ISH was carried out in all EBV positive NAG samples, clear evidence of EBV infection of epithelial cells was found in two samples; in one of the samples the signal seemed to originate exclusively from epithelial cells (Figure 5d), while in the other NAG the signal was equally prominent in epithelial and mononuclear cells (see Table 2). This latter sample also showed the largest infiltrate of mononuclear cells of all positive NAGs. In the remaining four NAG samples EBV positive cells were observed scattered in the lymphoid infiltrate of the lamina propria. Two EBV negative GCs, two EBV negative NAGs and three bypasses (both samples with follicular gastritis and one with metaplasia) were EBER1 stained. No EBV positive signal was found in any of the those samples (Figure 5 and Appendix Figure A1)

An immunohistochemistry (IHC) staining of cytokeratin was used to better identify epithelial cells. Examples of EBV positive and negative GC and NAG samples are shown in Figure 5. 

### 2.8. Histopathological Findings

**Figure 6:**
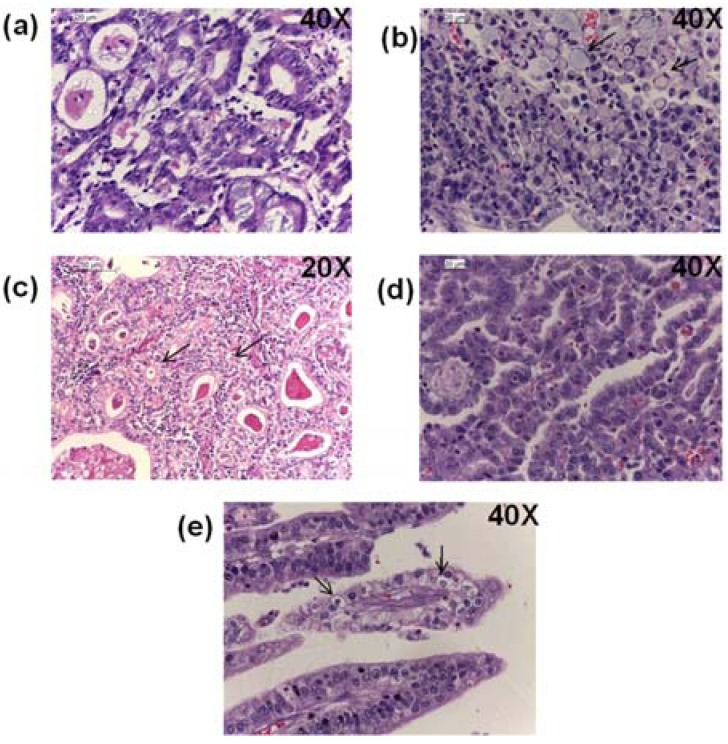
Histological patterns of the EBV positive gastric cancers. Typical gastric adenocarcinomas according to Lauren’s classification are shown: (**a**) Intestinal, and (**b**) diffuse types, with black arrows pointing to the characteristic “signet ring cells”. Gastric adenocarcinomas patterns found in the EBV positive samples are shown: (**c**) intestinal type with cribriform pattern and clear cells (arrows), (**d**) intestinal type with lace pattern and (**e**) mixed intestinal and diffuse with clear cells (arrows) present in the intestinal component.

Three gastric tumors were located in the body, one of which was infiltrating through the gastric wall and the serosa layer, two were in the antrum and three extended in both body and antrum (Table 1). Among the eight EBV positive GC samples, five (62.5%) were of intestinal type (Figure 6a), two (25%) were diffuse (Figure 6b) and one (12.5%) was of a mixed type. Of note, five positive samples presented additional GC histological patterns: three samples showed a cribriform pattern, one of which also showed large clear cells (Figure 6c) and two showed the previously EBV-associated *lace pattern* (Figure 6d). One of the EBV positive GCs also showed a large immune cell infiltrate resembling a LEL type but without syncytia formation and with polymorphonuclear cells in addition to the typical mononuclear cells; therefore, it was not considered a LEL type. This latter GC also showed large clear cells. Forty-five EBV negative GCs were re-examined to search for these patterns and none of them showed LEL, cribriform, or large clear cells, arguing for an enrichment of these patterns in EBV positive GCs.

Severe mononuclear cell infiltration was observed in four of the EBV positive NAG samples with only mild infiltration found in the remaining two. A moderate infiltration of polymorphonuclear cells was observed in three samples, all of them from the body of the stomach, and no activity was observed in the remaining three NAGs from the stomach antrum (Table 2). Three out of five EBV positive NAGs presented *H. pylori* colonization, two were negative and information was not available from one of the NAGs. No evidence of clinicopathological differences were found between EBV positive and negative NAGs, or between NAGs with epithelial or mononuclear cell infection.

### 2.9. Discussion

Our results support a role of EBV in 10.67% of the GC cases; a similar world-wide frequency has been reported in a recent meta-analysis [7]. Geographical variation in the frequency of EBV positive GC samples has been observed. A couple of pioneering studies had previously addressed the frequency of EBV infection in GC from Mexican patients [14,15]. We observed a higher frequency of positive cases than in the above studies, 10.67% *vs.* 7%–8%, and this difference is even greater if we consider that in both early reports, samples of LEL GC were included. Excluding LEL cases, those reports would support an EBV frequency of 4.6% in common types of GC. Frequencies of EBV positive GCs ranging from 17%–21% have been reported for Chile, Colombia and Peru [16,17,18]. On the other hand, our results are more similar to the frequencies reported for Brazil (11.2%–11.3%) [19,20].

EBV positive GC has been associated with different clinico-pathological features. A recent meta-analysis showed that young patients are more likely to have EBV positive GCs, but only for male patients and no age preference was observed for women [7]. With respect to gender, one article observed a female predominance [21], but most reports found a 2-fold higher male predominance [8]. Also, EBV positive GCs are more often originated in the proximal region of the stomach. We did not observe an obvious age, gender or stomach region preference in the EBV positive tumors (see Table 1); however, due to the low number of positive cases we were unable to do a logistic regression analysis. In regard to the Lauren GC type, it has been proposed that EBV-associated GCs are almost twice as likely to be diffuse [8]. We observed that the majority of EBV positive cases were of intestinal type. Studies in which LEL GCs are included often support an EBV association with stomach body and cardia, males and Lauren’s diffuse type. We purposely excluded LEL (also considered of good prognosis) and stump GCs, which are highly associated with EBV [22,23,24,25]. Future studies should differentiate those GCs to better understand EBV involvement in the most common types of GC.

A distinctive histological pattern has been described for EBV positive GC samples, the so called *lace pattern*, characterized by connection and fusion of the neoplastic glands [26]. In this study, two out of eight positive samples presented this pattern. Considering that it was not found in a representative sampling of the EBV negative tumors, this study also supports that the *lace pattern* could be used as a biomarker for EBV-associated GC. It is presently unclear through what molecular mechanism EBV infection leads to acquisition of this characteristic pattern. A cribriform histological pattern was found in three of the eight positive samples. This is also a rare histological pattern in gastric adenocarcinoma, often referred to as gastric carcinoma with cribriform component (CGA) [27]. To our knowledge, no previous studies have associated this pattern in EBV positive GC. Importantly, one study found a worst prognosis for CGA than GC without a cribriform component [27]. We also found large clear cells in two EBV positive tumors (one lace pattern and one cribriform); this type of cell is common in carcinomas of the female reproductive organs and kidneys. We did not find any of these patterns in 45 of the EBV negative GCs. Although these results are interesting, whether EBV-associated GC is also related to a cribriform pattern and/or present large clear cells needs the analysis of a larger number of positive samples. 

Two EBV positive samples were found in non-tumor control tissues with the first PCR. GC sometimes spreads under the lamina propria and often tumor cells are present in areas adjacent to the tumor. Although, in this study all non-tumor controls were at least 2 cm apart from the tumor borders and were analyzed for the presence of tumor cells by microscopy to prevent false positives, the fact that both of these EBV positive control tissues were from patients with EBV positive tumors, suggests that tumor cells were contaminating adjacent tissues. Alternatively, EBV may also be infecting areas adjacent to the tumor in those control tissues. Although rare, similar results have been previously reported in hyperplastic or dysplastic tumor-surrounding tissues [14,28]. 

In this analysis, to consider an EBV positive signal the frequency of EBV infected B lymphocytes should be at least >62-fold (for GC) and >2-fold (for NAG) higher than the signal found in tumor tissues in which EBV infection is harbored in circulating B cells [10]. Also, both PCRs used in this analysis were designed above the level of EBV infection in peripheral mononuclear cells from healthy donors. When EBER1 ISH was performed, all GC samples tested and two NAG samples showed clear evidence of epithelial cell infection, while the remaining four positive NAG samples only showed infection of B cells. In *H. pylori*-associated gastric lesions, the inflammatory response plays a critical role in GC progression. Considering that our data argues for increased levels of EBV infection in the gastric mucosa in all NAG positive patients, it is possible that EBV is an important trigger of inflammation driven tissue damage. In agreement, we have previously found that EBV infection is necessary to develop severe gastritis in pediatric patients [9], and we observe similar results in pre-neoplastic lesions from adult patients (unpublished data).

Only a handful of studies have previously addressed the presence of EBV in gastritis, and they have mainly analyzed atrophic gastritis arguing that it is closer to the cancer stage [29,30,31,32,33,34]. We decided to analyze non-atrophic gastritis because of the studies supporting a preferential role of EBV in diffuse GC, and NAG is the starting stage for both diffuse and intestinal GC [4]. The above mentioned studies have found heterogeneous results: from no evidence [29] or low frequency of EBV infection (3.0%) [30], to frequencies higher than the one found in this study [31,32]. There is also more indirect evidence supporting an EBV role in gastritis, such as elevated anti-EBV antibody titers [33], and a case report in which progression from gastritis to GC was observed in a post-transplanted *H. pylori* negative patient [34]. Taken together, all these data suggest that it is possible that EBV participates in the progression to GC by both direct and indirect mechanisms: (1) by infecting epithelial cells, establishing a latent program in which viral oncogenes are expressed, and (2) by favoring chronic inflammatory responses leading to tissue damage because of augmented local viral loads. Interestingly, although *H. pylori* is the best known model of inflammation-triggered transformation, the bacteria also harbors the CagA oncoprotein whose sole expression induces formation of GI tract carcinomas and lymphomas in transgenic mice [35,36]. It is possible that direct expression of oncogenic products, together with infection-triggered inflammation are the causal mechanism of transformation of the gastric mucosa, similarly to HBV and HCV induced hepatocellular carcinoma [37,38]. However, we cannot rule out that EBV is an innocent bystander in gastritis tissue and its presence is mainly due to the inflammatory response attracting infected B cells. 

## 3. Experimental Section

### 3.1. Cell Lines

EBV positive cell lines Raji, Daudi and B95-8 and EBV negative cell lines Ramos and BJAB were cultured in RPMI advance medium complemented with 4% fetal bovine serum (FBS) and Hepes 1× (RPMI, FBS and Hepes are from Gibco Invitrogen, Grand Island, NY, USA) and maintained in 5% of CO_2_ at 37 °C. EBV negative MCF10A epithelial cells were cultured in DMEN/F-12 medium supplemented with 20 ng/mL of epidermal growth factor (PeproTech, Rocky Hill, NJ, USA), 10 μg/mL insulin, 0.5 μg/mL hydrocortisone, 100 ng/mL cholera toxin (all from Sigma Chemical Co., St. Louis, MO, USA), and 5% fetal horse serum (Gibco Invitrogen, Grand Island, NY, USA).

### 3.2. Patients and Samples

The tissues analyzed in this study were: tumor, non-tumor tissue isolated from the gastric resection of a cancer patient (referred as non-tumor controls), gastric biopsies from non-atrophic gastritis (NAG) and gastric bypass surgery samples. All non-tumor controls were at least 2 cm apart from the tumor borders. Patients were recruited from two different cities with a high incidence of GC: Mexico City (mostly urban) and Merida Yucatan (mostly rural) (see Table 3). In Mexico City all patients with GC were enrolled prospectively; All NAG biopsies and GC samples from Merida were retrospective cases. Tissue samples from Mexico City were frozen at −70 °C immediately after surgical removal. Samples from Merida were already embedded in paraffin and DNA was extracted from paraffin slides. The following cases were recruited from Mexico City: 44 GC with their paired 44 non-tumor control, 11 GC without their paired non-tumor tissue, 99 non-tumor controls from which tumor tissue was not available, and 48 patients with NAG. Patients included from Merida were: 20 patients with GC in which only tumor tissue was available, 4 non-tumor controls and 27 NAG. 19 samples were obtained from an equal number of patients that underwent gastric bypass surgery because of obesity related problems. These patients were from the Bernardo Sepulveda Specialties Hospital in Mexico City and were paraffin embedded tissues retrospectively collected. All of these amount to a total of 316 samples analyzed: 75 GCs, 147 non-tumor controls, 75 NAGs and 19 gastric bypasses. Table 3 describes GC and NAG samples and patients included in the study. 

**Table 3:** Summary of samples: origin and type.

Hospital	Gastric Sample	Gender		Mean Age	Condition
		Male *	Female *		
GC Mexico City: Oncology Hospital, General Hospital and National Institute of Cancer	34 Diffuse	23	31	61	Fresh and frozen
	17 Intestinal				
	4 Mixed				
	44 Non-tumor controls				
GC Merida, Yucatan: Ignacio Garcia Tellez Hospital.	10 Diffuse	9	11	62.6	Paraffin embedded
	10 Intestinal				
Non-tumor controls, Mexico City: Bernardo Sepulveda Specialties Hospital, Oncology Hospital, General Hospital, National Institute of Cancer and Carlos McGregor Hospital	99 Non-tumor controls	43	43	61.6	Frozen
Non-tumor controls Merida Yucatan: Ignacio García Tellez, Hospital	4 Non-tumor controls	2	2	71.5	Paraffin embedded
Non-atrophic gastritis, México City: Bernardo Sepulveda Specialties Hospital and Carlos McGregor Hospital.	49 NAG	26	23	49.5	Frozen
Non-atrophic gastritis, Merida Yucatan: Ignacio Garcia Tellez Hospital	27 NAG	10	17	52.6	Paraffin embedded

GC = gastric cancer samples. * We were not able to obtain the gender of one of the GC patients and 13 of the non-paired non-tumor controls.

### 3.3. Histopathological Examination

A fragment of all tissues was fixed in formaldehyde and embedded in paraffin, a slide was stained with hematoxylin-eosin (HE) and analyzed by an experienced pathologist to determine the type and grade of the lesion. Tumor samples were diagnosed according to Lauren’s and WHO’s criteria and the frequency of tumor cells was estimated; all tumor tissues included in the study had ≥5% of tumor cells. The tumor and NAG samples were classified by the updated Sydney system according to the infiltrate of immune cells (mononuclear and polymorphonuclear) and the presence of *H. pylori*. A single pathologist with experience in the Sydney classification performed this analysis.

### 3.4. DNA Purification

DNA was obtained from all EBV positive and negative lymphoma cell lines, peripheral blood leukocytes, frozen and paraffin-embedded tissues, using Qiagen DNA extraction kits (Qiagen, Hilden, Germany) following the manufacturer instructions. For cell lines, DNA was obtained from 5 million cells using the QIAamp mini kit. For blood leukocytes, 8 mL of blood were drawn from healthy donors, placed in CPT cell preparation tubes (BD Vacutainer, Franklin Lakes, NJ, USA), a million cells were subjected to DNA extraction using the QIAamp mini kit. For frozen tissues, up to 10 mgs were disrupted in a TissueLyser II (Qiagen, Hilden, Germany) for 20 s and homogenates were subjected to DNA purification with QIAamp DNA mini kit. For paraffin-embedded tissues; sections of 5–10 µm thick were cut, and DNA was purified using the QIAmp DNA FFPE tissue kit. Purified DNA was quantified using a spectrophotometer NanoDrop 1000 (Thermo Fisher Scientific, Waltham, MA, USA) and DNA quality was determined with the 260/280 ratio, integrity by electrophoresis in agarose gels and by PCRs of a short β-globin (167 bp) and a long β-actin (670 bp) fragments of endogenous genes [39].

### 3.5. EBV Detection

DNA samples were subjected to a first PCR with primers LLW1 y LLW2 [40], which amplify a region within the BamHI W fragment repeated *in tandem* in the EBV genome [41]. The PCR mix (50 μL) contained 200 ng of template DNA, 200 μM of dNTP mix, 2.5 mM of MgCl_2_, 5 μL of Taq Polymerase buffer 10× with (NH_4_)_2_SO_4_, 200 nM of each primer, and 2.5 U of Taq Polymerase (buffer and polymerase from Thermo Fisher Scientific, Waltham, MA, USA). The PCR reaction was: an initial denaturation step of 5 min at 94 °C and then 30 cycles of 94 °C for 1.5 min, 57 °C for 45 s and 72 °C for 1 min, and a final extension of 72 °C for 7 min. 

Internal primers to the first PCR amplicon were designed for the nested PCR: LLWint1 5'-CTT TGT CCA GAT GTC AGG GG-3' and LLWint2 5'-GCC TGA GCC TCT ACT TTT GG-3'. The 50 μL PCR mixture contained 1 μL of a 1/1,000 dilution of the first PCR, 200 μM of dNTPs mix, 2.5 mM of MgCl_2_, 5 μL of Taq Polymerase buffer 10× with (NH_4_)_2_SO_4_ (Thermo Fisher Scientific), 400 nM of each primer, and 2.5 U of recombinant Taq Polymerase (Thermo Fisher Scientific). The reaction was performed with an initial denaturation step at 94 °C for 5 min, followed by 15 cycles of 94 °C for 20 s, 57 °C for 20 s and 72 °C for 30 s, and a final extension of 72 °C for 7 min. 

### 3.6. Sequencing

Positive samples were confirmed by sequencing of both forward and reverse strands of the PRC products (purified with QIAquick gel extraction kit, Qiagen, Hilden, Germany). Sequences were compared with the Genebank database using the BLAST program [42]. Sequencing was carried out in the Instituto de Biología, Universidad Nacional Autónoma de México.

### 3.7. *In Situ* Hybridization

ISH was performed with the probe: 5'Biotin-AGACACCGTCCTCACCACCCGGGACTTGTA-3' for EBER1 as previously used by Luo *et al*. [43]. Samples were incubated with the probe overnight at 37 °C and detection was carried out with Dako *In Situ* Hybridization Detection System For Biotinylated Probes (Dako, Inc., Carpinteria, CA, USA), using an astringent temperature of 51 °C.

### 3.8. Immunohistochemistry (IHC)

IHC for cytokeratin was performed with anti-human/mouse cytokeratin AE1/AE3 cocktail antibody (Biocare Medical, Concord, CA, USA). Endogenous peroxidase activity was blocked by incubating the slides in peroxidase blocking solution (Dako, Inc., Carpinteria, CA, USA). Nonspecific antibody binding was blocked incubating 30 min with Power Block Universal Blocking Reagent (BioGenex, San Ramon, CA, USA). Cytokeratin AE1/AE3 cocktail antibody was incubated at 1:50 concentration one hour at room temperature and detection of the staining reaction was achieved with the Envision Detection Kit (Dako, Inc., Carpinteria, CA, USA). Samples were counterstained with hematoxylin. 

### 3.9. Statistical Analysis

The increased probability of finding EBV in GC samples than in non-tumor controls was estimated using the odds ratio (ORs) and 95% confidence intervals (CIs). 

## 4. Conclusions

EBV positive results were found for 10.67% of GC and 8% of NAG samples. Since our data support an augmented frequency of EBV infected cells in epithelial cells in GC and in epithelial and B cells in NAG, it is possible that EBV contributes to GC progression by both direct oncogene expression and indirect inflammatory triggered tissue damage mechanisms. 

## Appendices

**Table A1:** Characteristics of samples from gastric bypass surgery.

Muestra	*H.pilory*	PMN cells	MN cells	Atrophic Gastritis	Metaplasia	Dysplasia	Additional observations
SGB 001	+	0	++	0	0	0	Follicular gastritis
SGB 002	+	0	++	0	0	0	Follicular gastritis
SGB 003	0	0	+	0	0	0	None
SGB 004	+	0	++	0	0	0	None
SGB 005	0	0	+	0	0	0	None
SGB 006	0	0	+	0	0	0	None
SGB 007	+	+	++	0	0	0	None
SGB 008	0	0	0	0	0	0	None
SGB 009	0	0	+	0	0	0	None
SGB 010	0	0	++	0	0	0	None
SGB 011	0	0	+	0	0	0	None
SGB 012	0	0	+	0	0	0	None
SGB 013	0	+	++	0	0	0	None
SGB 014	0	0	+	0	0	0	None
SGB 015	0	++	+++	+++	++	0	Neuroendocrine cell hyperplasia
SGB 016	0	0	+	0	0	0	None
SGB 017	0	0	+	0	0	0	None
SGB 018	0	0	++	0	0	0	None
SGB 019	0	0	0	0	0	0	None

0 to +++ = none, mild, moderate and severe. PMN and MN = polymorpho and mononuclear cell infiltrate.
